# An Overview of Challenges Limiting the Design of Protective Mucosal Vaccines for Finfish

**DOI:** 10.3389/fimmu.2015.00542

**Published:** 2015-10-22

**Authors:** Hetron Mweemba Munang’andu, Stephen Mutoloki, Øystein Evensen

**Affiliations:** ^1^Section of Aquatic Medicine and Nutrition, Department of Basic Sciences and Aquatic Medicine, Faculty of Veterinary Medicine and Biosciences, Norwegian University of Life Sciences, Oslo, Norway

**Keywords:** bath, antigens, finfish, genomics, immersion, mucosal, oral, vaccines

## Abstract

Research in mucosal vaccination in finfish has gained prominence in the last decade in pursuit of mucosal vaccines that would lengthen the duration of protective immunity in vaccinated fish. However, injectable vaccines have continued to dominate in the vaccination of finfish because they are perceived to be more protective than mucosal vaccines. Therefore, it has become important to identify the factors that limit developing protective mucosal vaccines in finfish as an overture to identifying key areas that require optimization in mucosal vaccine design. Some of the factors that limit the success for designing protective mucosal vaccines for finfish identified in this review include the lack optimized protective antigen doses for mucosal vaccines, absence of immunostimulants able to enhance the performance of non-replicative mucosal vaccines, reduction of systemic antibodies due to prolonged exposure to oral vaccination and the lack of predefined correlates of protective immunity for use in the optimization of newly developed mucosal vaccines. This review also points out the need to develop prime-boost vaccination regimes able to induce long-term protective immunity in vaccinated fish. By overcoming some of the obstacles identified herein, it is anticipated that future mucosal vaccines shall be designed to induce long-term protective immunity in finfish.

## Introduction

Mucosal vaccination has emerged to be one of the major areas in fish vaccinology that has attracted a lot of research in recent years. This has been exacerbated by the increasing demand for less labor-intensive vaccination strategies as an alternative to the strenuous injectable vaccines that require individual handling of fish that may result in stress-related immunosuppression and handling mortalities. Mucosal vaccination is less labor-intensive given that mucosal vaccines are administered by immersion, oral, or bath without individual handling of fish. Mucosal vaccination is of particular value where prime-boost vaccination strategies are preferred typically with a combination of primary injection followed by mucosal boost administration. Mucosal vaccines outweigh injectable vaccines from a practical viewpoint in fish, this far, there are no licensed mucosal vaccines shown to produce superior protection over injectable vaccines except for the live-attenuated Cyprinid herpesvirus 3 (CyHV-3) vaccine administered by immersion in carp (*Cyprinus carpio*) in Israel (1, 2). There is a need to better understand factors that limit the efficacy of mucosal vaccines for finfish and in this review, we bring into perspective some of the major challenges hindering the design of protective mucosal vaccines for finfish.

## Choice of Antigen Delivery Systems for Mucosal Vaccines in Finfish

One of the major challenges hindering our progress in developing highly protective mucosal vaccines for fish is the choice of antigen delivery system. Table 1 shows a comparison of different antigen delivery systems for mucosal vaccines in finfish. The major factors that influence the choice of antigen delivery system include (i) safety (for live reversion to virulence), (ii) ability to evoke a strong innate and adaptive immune response in vaccinated fish, (iii) for oral vaccines, the ability to deliver vaccines to the second gut segment without denaturation in the acidic stomach environment, (iv) ability of antigens to cross mucosal barriers in order to gain access to antigen presenting cells (APCs) for induction of local and/or systemic responses, (v) and to correlate the administered antigen dose with protective immunity. These elements constitute some of the major factors that influence the optimization of antigen delivery system used for fish mucosal vaccines.

**Table 1:** **Comparison of different antigen delivery systems for mucosal vaccines for finfish**.

Antigen delivery system	Safety	Immunogenicity	Induction of innate immunity	Induction of adaptive immunity	Reference
**(A) Live vaccines**					
Attenuated CyH-3 vaccine	Low	+++	+++	+++	(2)
Attenuated VHSV vaccine	Low	+++	+++	+++	(3)
Recombinant live virus protein	High	+++	+++	+++	(4)
**(B) Inactivated vaccines**					
Inactivated whole pathogen	High	++	++	++	
Subviral particle vaccines	High	+	+	+	(5)
Subunit vaccines	High	+	+	+	(6)
**(C) Encapsulations/top dressing**					
PLGA nanoparticle vaccine	High	+	+	+	(7, 8)
Chitosan nanoparticle vaccine	High	+	+	+	(9)
PLGA microparticle vaccine	High	+	+	+	(10)
Chitosan microparticle vaccine	High	+	+	+	(11)
Alginate vaccines	High	+	+	+	(12)
Micromatrix vaccines	High	+	+	+	(13)

Antigens for fish mucosal vaccines can be replicative or non-replicative antigens (14). The replicative antigens comprise of live-attenuated pathogens and in the broadest sense, DNA vaccines. The only live mucosal vaccine licensed for fish is the CyHV-3 vaccine used for the vaccination of carp (1, 2, 15). Some of the factors that hinder the use of live vaccines in fish include the fear of reversion to virulence as shown in the case of infectious pancreatic necrosis virus (IPNV) where avirulent strains can revert to virulence under stress conditions (16). Another important factor is the fear of introducing live pathogens in the aquatic environment, which could become pathogenic to other species. Unlike live vaccines that replicate in infected cells, DNA vaccines use the host cell machinery to transcribe their antigenic proteins *in vivo*. The major concern with DNA vaccines is the fear of the vaccine’s DNA being integrated into host cellular genome, which could alter the cells’ replication mechanisms. To date, a DNA vaccine against infectious hematopoietic necrosis (IHN) has been licensed in Canada (17). Both live and DNA vaccines evoke humoral and cellular-mediated immune (CMI) responses in vaccinated fish, less so for DNA vaccines. They induce long-term protection and adjuvants are not needed for induction of immunity.

Non-replicative vaccines include killed pathogens, their protective antigens/epitopes in native or synthetic form, or less well-defined structural components of the pathogen (18). They will usually not evoke CMI responses, and elicited responses are biased toward humoral immunity (18). They require the help of adjuvants to enhance their immunogenicity and boost vaccination to maintain long-term protective immunity. Despite so, non-replicative vaccines constitute the biggest bulk of vaccines currently used in aquaculture because of their safety (18). Therefore, there is need to optimize the performance of these antigens in order to develop highly protective mucosal vaccines for finfish.

As shown in Table 1, live and DNA vaccines are classified as more immunogenic than the non-replicative vaccines (14). This is mainly because these vaccines replicate at the site of antigen deposition inducing prolonged inflammatory responses that attract APCs resulting in presentation to immune cells of the adaptive immune system. On the other hand, vaccine-induced activation of APCs is limited in the absence of antigen replication for non-replicative vaccines. Hence, the replicative vaccines have a higher capacity to induce innate and adaptive immune responses than the non-replicative vaccines (14).

## Choice of Adjuvants for Mucosal Vaccines

In order to enhance the immunogenicity of non-replicative vaccines, there is a need to develop mucosal adjuvants for incorporation in vaccine formulations. Adjuvants have a dual function serving as (i) antigen delivery vehicles and (ii) immunostimulants (19, 20). As antigen delivery vehicles, adjuvants are designed to serve as a depot to allow for slow release of antigens so as to generate long-term protective immunity. Developing adjuvants that would form depots on mucosal surfaces might not be feasible in fish. Hence, there is a need to devise vaccination strategies with an optimal duration of exposure to allow for adequate antigen uptake in mucosal organs. Immunostimulants activate APCs to carry out antigen uptake, processing, and presentation to cell of the adaptive immune system. In a recent review (21), we have shown that all mucosal organs in finfish are endowed with APCs, such as monocytes, macrophages, and dendritic-like cells. We have also reviewed the mechanisms of antigen uptake and presentation in finfish and showed that fish APCs have similar ligands that bind to B- and T-lymphocytes with mammalian APCs (14). In addition, fish APCs are responsive to different cytokines and chemokines based on mechanism similar to mammalian APCs suggesting that fish APCs can be activated by immunostimulants to enhance antigen uptake and presentation in a similar way (14, 21).

Immunostimulants explored for mucosal vaccine delivery in finfish include poly(lactic-co-glycolic acid) (PLGA) nanoparticles (7, 8), microparticles (10), micromatrix-particles (13), and alginates (12, 22) that also serve as antigen delivery vehicles. Other immunostimulants explored include TNFα (23), IFNα (24), CpG (25), β-glucan (26, 27), and lipolysacharride (LPS) (26, 27). However, the extent to which these immunostimulants enhance antigen uptake in mucosal organs has not been investigated in detail in finfish. In mammals, different mucosal adjuvants have been developed of which the most promising are those made from heat-labile enterotoxin bacteria. The cholera toxin subunit B (CTB) adjuvant is one of the most potent and only mucosal adjuvant incorporated in licensed vaccines in humans (28). It has been shown to increase the level of mucosal IgA responses by four to sixfold and systemic IgG levels by 250-fold for intranasal mucosal vaccines (29–31). Although mucosal vaccines are designed to increase IgT and IgM levels in finfish (32), there are no studies shown to increase the levels of mucosal antibodies based on inclusion of adjuvants in mucosal vaccine formulations. There is need to develop immunostimulants able to enhance the immunogenicity of non-replicative mucosal vaccines in finfish.

## Optimization of Antigen Dose in Mucosal Vaccine Design for Finfish

As pointed out by Neutra and Kozlowski (33), vaccine antigens deposited on mucosal surfaces face the same gauntlet of host defensins as microbial pathogens. They are diluted or washed away in mucus, degraded by antimicrobial compounds or excluded by epithelial barriers. For oral vaccines, antigens can be denatured by the acidic environment in the stomach or digested in the foregut such that they might not reach the mid-intestine/hindgut in an immunogenic form. As a result, it is difficult to determine the exact dose that reaches the compartments believed to play in important role in immune induction locally or to what extent antigens cross the mucosa barriers, which makes it difficult correlate the antigen dose with immune protection for mucosal vaccines. For the antigens to cross mucosal barriers, it is anticipated that they should mimic the native pathogens and thus retain the ability to translocate/penetrate mucosal surfaces (33). These attributes entail that live-attenuated vaccines that use similar strategies as the target pathogen to induce local responses and/or penetrate mucosal barriers are the best candidates for mucosal vaccination. As pointed out above (see Choice of Antigen Delivery Systems for Mucosal Vaccines in Finfish), live vaccines are less used in aquaculture because of safety reasons. Therefore, the challenge is to develop antigen delivery systems able to increase the antigen dose that crosses mucosal barriers for non-replicative vaccines.

To enhance antigen uptake through mucosal surfaces, different immunization strategies have been explored in mammals. For example, Chen et al. (34) developed a nanoparticle vaccine delivery system by incorporating anionic liposomes into chitosan/DNA complexes, which enhanced antigen uptake by increasing the residence time of antigens on nasal mucosal surfaces resulting in high absorption efficiency. In addition, these particles had a high DNA-antigen loading efficiency and effective protection against nucleases. Srinivasan and Burgess (35) have pointed out that the use of anionic lipids, which are natural component of eukaryotic cells, in micro- and nanoparticle formulations increases the adsorption capacity of antigens through mucosal surfaces resulting in high transfection efficiency in host cells. Another approach being explored is the use of replicon vectors of which the most commonly explored are alphavirus replicon vectors (36, 37). Alphavirus-based replicon vectors lack the viral structural protein genes, which render them replication incompetent and non-lethal. However, they maintain the replicative elements necessary for amplification of the inserted heterologous immunogenic protein via an active alphavirus promoter (38). The advantage of using such delivery systems is that the replicon harboring the RNA vector has a tropism for different cell types enabling the exploitation of *in vivo* targeting of APCs to enhance antigen uptake (39). It is possible to use some of the strategies used to enhance antigen uptake through mucosal barriers in mammals in the design of mucosal vaccines for finfish. These avenues have only been explored to a very limited extent so far.

## Route of Mucosal Vaccine Delivery in Finfish

In fish, there are three routes by which vaccines are administered namely the immersion, oral, and injection routes. As shown in Table 2, the injection route is labor intensive as it requires individual handling of fish, which can lead to stress-related mortalities. On the other hand, the immersion route is less labor-intensive and mimics natural exposure to infection. Although the immersion route is easy to apply in small fish reared in tanks, it is not easily applicable for large fish in open cages at sea. The oral route is less labor-intensive and is, in principle, applicable at all stages of fish production. However, the advantage for the injection route is that it is possible to quantify the antigen dose that correlates with protective immunity (40), which is not easy to quantify for the oral and immersion routes. Nakanishi et al. (41) have pointed out that for immersion vaccination, antigen uptake was highly influenced by the duration of exposure to the vaccine, total biomass, age, pH, and salinity of the water used for immersion. In the case of oral vaccination, antigen uptake is influenced by the pH in the gut, appetite of the fish, absorption through mucosal barriers, and several other undetermined factors. These elements suggest that antigen dose optimization for the oral and immersion vaccination routes might not be easily attainable. The injection route is the only route that has been shown to easily correlate the antigen dose administered in fish with protective immunity (40). This would account for the reasons why the largest bulk of vaccines currently used in aquaculture are injectable (14).

**Table 2:** **Comparison of different routes of mucosal vaccine delivery in finfish**.

Parameter	Oral delivery	Immersion delivery	Injection delivery
Efficacy	Low	Low/moderate	High
Labor input	Low labor input	Low labor input	Intensive labor input
Size of fish	Unlimited	Unlimited	Limited
Individual handling of fish	No	No	Yes
Open cages in seawater	Highly applicable	Not easily applicable	Not easily applicable
Closed system/cages in freshwater	Highly applicable	Highly applicable	Applicable
Stress	Low	Low	High
Route of exposure	Gut/intestine	Natural portals of entry	Parenteral

## Measures of Efficacy and Correlates of Protective Immunity for Mucosal Vaccines

In mammals, the licensing of most vaccines is based on established correlates of protective immunity (42–44). A commonly used correlate of protection in mammals is antibody levels expressed in response to vaccination (44). Predetermined antibody titers serve as correlates of protective immunity for which once a vaccine dose attains the established cutoff limit, it is considered potent. Although vaccine development has been going on in fish vaccinology for a long time, data on the correlates of protection for licensed vaccines are not available in published journals. Hence, the cutoff limit of antibody responses at which mucosal antibodies can be considered protective in vaccinated fish are not established for mucosal vaccines in finfish. Studies carried in finfish show that mucosal vaccines induce both mucosal and systemic antibody responses in vaccinated fish (4, 21). The role of mucosal antibodies is to protect mucosal sites and prevent the pathogen from entering systemic distribution through mucosal linings. Moreover, it has been shown that there is compartmentalization in the functional roles of mucosal antibodies with IgT being predominantly found on mucosal surfaces, while IgM is found in circulation and likely plays a more important role in preventing systemic pathogen dissemination (21). Therefore, to correctly ascertain the protective ability of mucosal vaccines, protection should be measured in the context of determining the protective role of (i) mucosal antibodies at mucosal surfaces, (ii) systemic antibodies in the systemic environment, and (iii) their individual and/or combined function in reducing or preventing post challenge mortality in vaccinated fish.

### Protection Against Pathogen Entry at Mucosal Surfaces

This component of protective immunity is mostly centered on preventing the establishment of infection in vaccinated fish. Studies in mammals have shown that the expression of secreted IgA correlates with resistance to infection (45–47). It has been shown that secreted IgA in mucus has the capacity to neutralize viruses and prevent their adherence to epithelial surfaces (45–48). Given that IgT is the most predominant Ig isotype found on mucosal surfaces in finfish (49–52), it plays a role in preventing pathogen attachment and localization possibly using mechanisms similar to those seen for IgA in mammals. In finfish, there are no established quantitative assays for measuring IgT titers in mucus. Thus, it is difficult to determine the threshold level of IgT able to deter pathogen attachment, colonization, and entry at mucosal surfaces. And as such, it is difficult to determine the antigen dose of mucosal vaccines able to induce IgT titers that would serve as correlates of protection against infection and/or prevention of pathogen entry through mucosal surfaces. No doubt, there is a need to develop a quantitative assay for IgT in mucus in order to pave way into establishing correlates of protection.

### Protection Against Pathogen Dissemination to Systemic Organs

This component of protective immunity is centered on preventing disease establishment by blocking pathogen dissemination to internal organs that include target organs that are prone to pathogen-induced pathology (40, 53). Although mucosal vaccines have been shown to produce systemic antibody responses (4, 54, 55), there are no studies shown to generate systemic antibody titers that would serve as correlates of protection for mucosal vaccines in finfish. Thus, it is difficult to define the antigen dose of mucosal vaccines that would induce protective antibodies against disease establishment. In our previous studies (40), we showed that antibody levels ≥1.2 OD_490_ (1:50 dilution) prevented the establishment of pathology in target organs in Atlantic salmon vaccinated against IPN. In another study (53), an antibody titer ≥1.4 OD_490_ (1:50 dilution) prevented against IPNV-induced pathology indicating that a threshold antibody titer can be established for vaccines against IPNV infection to serve as a cutoff limit at which protection can be defined. It is likely that similar approaches can be used to develop correlates of protective immunity for mucosal vaccines in finfish.

### Protection Against Mortality

Although it has been shown that mucosal vaccines have the capacity to reduce post-challenge mortality in vaccinated fish (4, 56–60), there are no studies that correlate the antibody responses induced by mucosal vaccines with post-challenge protection. In our previous studies, we showed that antibody titers ≥1.2 OD_490_ (1:50 dilution) corresponded with post-challenge survival proportions (PCSP) >92.0% in Atlantic salmon vaccinated against IPNV using injectable vaccines (40). In another study (53), antibody levels ≥1.4 OD_490_ (1:50 dilution) corresponded with PCSP >94.0%, again indicating that antibody titer that can serve as a correlate of protection for IPN vaccines in Atlantic salmon. Efforts should be made to determine similar correlates of protection for mucosal vaccines.

## Oral Tolerance to Mucosal Vaccination in Finfish

In mammals, studies on oral tolerance date as far back as the 1940s when Chase (61) showed that feeding a single chemical, such as picryl chloride, resulted in significant loss of systemic immune responses to subsequent exposure to the same protein due to a systemic delayed hypersensitivity reaction. This phenomenon of prolonged exposure to the same substance resulting in failure to induce systemic immune responses has become a gold standard test for oral tolerance (62, 63). In finfish, it has been demonstrated in Atlantic salmon (*Salmo salar* L.) (64, 65), rainbow trout (*Oncorhynchus mykiss*) (66), common carp (*C. carpio*) (67, 68), and gold fish (*Carassius auratus*) (69). In carp, it was shown that prolonged oral vaccination led to suppression of systemic antibody responses after administering the same antigen by injection (67, 68). Maurice et al. (69) exposed goldfish to prolonged oral vaccination against *Aeromonas salmonicida* A-layer recombinant protein and showed a decline in antibody levels after 1 month, while fish subjected to 5 days of oral vaccination had high antibodies. When fish in both groups were intraperitoneally injected with the same vaccine, only the group vaccinated for 5 days had an increase in systemic antibody levels that corresponded with high post challenge protection. In contrast, the group subjected to prolonged oral vaccination had insignificantly low systemic antibody levels linked to high post-challenge mortality. These studies suggest that prolonged oral vaccination in finfish could lead to decrease in systemic antibody responses likely caused by oral tolerance. The challenge is to find an optimal duration of oral vaccination that does not cause a reduction of systemic antibody responses in orally vaccinated fish.

In mammals, factors linked to immunotolerance include direct inactivation of antigen-sensitized lymphocytes via depletion or anergy, regulatory T-cells (T-regs), hepatic processing of antigens, and several other factors that include anti-idiotypes (47, 62, 70, 71). Tolerance can also be initiated by use of tolerogenic protein molecules in mucosal organs (62) of which none of the feed carriers, such as PLGA nanoparticles and alginates, used for oral vaccination in finfish have been tested for their tolerogenic properties. It has been shown that the expression patterns of some cytokines are prone to induction of tolerance, while others are not (62, 70). Factors that favor Th1 responses abrogate mucosal tolerance, while factors that favor Th2 and T-regs expression favor mucosal tolerance (62, 70), suggesting that the Th1/Th2 dichotomy can be used to monitor the progression of oral vaccination toward oral tolerance. Recently, Chen et al. (12) showed up regulation of GATA-3 a transcription factor for Th2 specification and FoxP3 a transcription factor for T-reg activation in Atlantic salmon orally vaccinated with inactivated, IPNV, which was suggestive of oral tolerance. Rombout et al. (72) reviewed the mechanisms of oral tolerance in fish and pointed out that most of the genes and cell types that regulate oral tolerance in mammals are present in fish. It is likely that the reduction of systemic antibodies linked to prolonged oral vaccination in finfish could be due to oral tolerance. There is a need to elucidate the underlying mechanisms linked to reduction of systemic antibody responses after prolonged oral vaccination in finfish.

## Impact of Species Variations on Mucosal Vaccine Design for Finfish

Fish can be clustered into different groups based on their physiological and immunological differences. They can also be classified into omnivores, carnivores, or herbivorous based on their feeding habits and anatomical layout of the digestive system. Rombout et al. (73) pointed out that 85% of the bony fish develop their stomach during the larvae stage, while cyprinids never develop a stomach rendering them lack a low pH environment in their digestive system. Stomached species degrade the vaccine antigens in the low pH of the gut unlike stomach-less species that lack a low pH environment in their digestive systems. For stomach-less species, the first segment of the gut is absorptive unlike stomached species that have a less absorptive first segment (73). Both stomach-less and stomached species have a high absorptive capacity in the second segment (73). These differences have a significant influence on the delivery of vaccines to the most absorptive parts of the gut. In stomached species, the target is to design vaccines able to survive the low pH in the stomach in order to reach the second segment of the gut. This implies that oral vaccines have to be tailor made for different species of which the use of encapsulated oral vaccines for stomached species would be ideal, while encapsulation might not be necessary for stomach-less species.

Another important factor that affects the design of protective vaccination strategies is the differences between cold and warm-water species. As pointed out by different scientists (74, 75), fish growth rate increases proportionally with increase in water temperature. Cold-water species, such as Atlantic salmon reared at 10–12°C, have a slow growth rate that require a long production period (~2 years, ≥7,300 degree days). On the contrary, warm-water species, like the Nile tilapia (*Oreochromis niloticus*), reared at 28°C have a shorter production period lasting about 6 months (≤6,000 degree days). It is likely that cold-water species could require multiple boost vaccinations to maintain high levels of protective antibodies during the long production periods, which might not be needed for fish species having short production periods. The difference between migratory and non-migratory species is another important factor that has a significant influence on the design of protective vaccination strategies. For example, Atlantic salmon undergo smoltification and changes in water salinity after transfer from freshwater to seawater making them vulnerable to stress-related disease outbreaks, such as IPNV outbreak (16, 76). Therefore, vaccination strategies should ensure that fish have protective antibodies at the stage when they are most vulnerable to outbreaks. Moreover, the transfer of fish from fresh to seawater calls for adjustments in vaccination strategies given that vaccination by injection is not practical for fish reared in open cages in seawater of which oral vaccination through feed is the most applicable strategy.

Difference on skin surface structures, such as the presence or absence of scales, is another important factor that could affect the efficacy of mucosal vaccines in different fish species. Scaled fish species are likely to have a low vaccine absorption capacity than scaleless species suggesting that optimization of the antigen dose required to induce protective immunity could be a greater challenge for scaled fish than scaleless fish. Another important factor influenced by species variations is the differences in immunological properties between different fish species. For example, Atlantic cod (*Gadus morhua*) lack genes that code for the MHC-II loci found in other fish species (77). Although it is not known how Atlantic cod compensate for the lack of MHC-II, it is likely that this accounts for differences in immune responses between Atlantic cod and other fish species that have the MHC-II genes. These differences indicate that it might not be practical to use a single vaccine for a disease that cuts across different fish species even when they are reared in the same ecosystem. Instead, different vaccines for the same disease should be tailor made for individual fish species requirements rendering vaccine production and optimization to be an expensive venture.

## Functional Genomics in Mucosal Vaccinology in Finfish

Recent advances in fish genomics have led to cloning and characterization of different genes that regulate different components of the innate and adaptive immune system in finfish. Genes of the innate immune system characterized in finfish include pattern recognition receptors (PRRs) that bind to pathogen-associated molecular patterns (PAMPs) on pathogens, surface markers of activated APCs and different regulatory cytokines and chemokines (14, 56, 57). Genes of the adaptive immune system characterized in fish include transcription factors that activate naïve T-cells into effector cells, T-cell receptors, CD3 receptors, and different regulatory cytokines of various T-cell responses (56, 57). Recently, we used RNA-seq to gain insight of the profile of genes expressed by macrophage/dendritic-like cells derived from Atlantic salmon head kidney leukocytes and showed that the repertoire of genes expressed by these cells were comparable to genes expressed by mammalian APCs (78). Therefore, the upcoming of high throughput genome mining techniques, such as RNA-seq, is expected to help identify more novel genes expressed in response to vaccination in finfish.

Given the lack of knock-out (KO) models and limited number of surface marker monoclonal antibodies (mAbs) characterized in finfish, a large proportion of immune response studies are dependent on gene expression. To a large extent, the interpretation of gene expression data in finfish is by inference to mammalian studies (53, 78, 79), where KO-models and abundance of surface marker mAbs have paved way to better understanding of immunological mechanisms of vaccine protection (80–84). In situations where no mammalian orthologs of genes identified in finfish exists, it is difficult to determine the functional roles of genes unique to fish in vaccine protection. In such situations, it is difficult to optimize vaccine performance based on gene expression when the functional roles of the target genes have not been characterized in vaccinated fish. Future studies should seek to use novel strategies, such as the morpholino oligonucleotide (85), transcription activator-like effector nuclease (TALEN) (86), and the clustered regularly interspaced short palindromic repeats (CRISPR) KO systems, to identify the functional roles of genes expressed in response to vaccination in finfish. By so doing, functional genomics could help identify genes that correlate with protective immunity for use in the optimization of mucosal vaccines for finfish.

## Prime-Boost Vaccination Strategies in Finfish

Prime-boost vaccination strategies can be classified into the homologous and heterologous vaccination strategies as shown below.

### Homologous Prime-Boost Vaccination Strategies

In situations where a single dose is insufficient to induce protective immunity, the same antigen can be administered repeatedly to increase and prolong the duration of protective immunity. This vaccination strategy has been widely used against different diseases in mammals (87), where the additive effect of repeated vaccinations increases immune responses to protective levels. In finfish, it has also been shown to increase antibody levels linked to reduction of post challenge mortality (Tables 3 and 4). In order to cope with different fish production strategies, homologous prime-boost vaccinations in finfish are in some cases carried out by administering the same antigens using different vaccine delivery routes. For example, in the case of Atlantic salmon primed by injection during the freshwater stage, oral boost after transfer to seawater in open cages is the most applicable strategy. In Table 3, the general trend is that homologous prime-boost vaccination regimes for the live vaccines are more protective than regimes for the inactivated vaccines irrespective of the route of vaccine delivery. For inactivated vaccines, the anal prime/anal boost vaccination strategy showed higher protection than the oral prime/oral boost regimes. Table 4 shows that the prime-boost regime for the viral hemorrhagic septicemia virus (VHSV) live vaccine had higher protection than the homologous prime-boost regimes using DNA vaccines. Another important factor shown to have a significant impact on the outcome of homologous prime-boost regimes is the antigen dose used in vaccination. For example, Kim et al. (4) showed a dose-dependent antibody response that corresponded with post-challenge protection in Olive flounder (*Paralichthys olivaceus*) vaccinated against VHSV using a homologous prime-boost vaccination strategy. Put together, these factors show that the efficacy of homologous prime-boost vaccination strategies is dependent on the route of vaccine delivery, type of antigen (live or inactivated), and antigen dose. In order to design protective homologous prime-boost vaccination protocols, it is important to find the most protective antigen delivery system, whether live or inactivated, antigen dose, and route of vaccination into fish for each disease and fish species.

**Table 3:** **Prime-boost vaccination strategies for bacterial diseases explored under experimental conditions in finfish**.

Disease	Prime delivery	Boost delivery	Fish species	Antigen	Protection	Reference
*Yersinia ruckei*	Immersion	Immersion	Rainbow trout	Live attenuated	Moderate	(54)
	Immersion	Injection	Rainbow trout	Live attenuated	High	(54)
	Immersion	Oral	Rainbow trout	Live attenuated	High	(55)
	Immersion	Bath	Rainbow trout	Live attenuated	Low	(54)
	Oral	Oral	Rainbow trout	Inactivated	Moderate	(88)
	Anal	Anal	Rainbow trout	Inactivated	High	(88)
*Aeromonas salmonicida*	Bath	Bath	Rainbow trout	Inactivated	Moderate	(89)
	Anal intubation	Anal intubation	Rainbow trout	Inactivated	High	(89)
	Injection	Oral	Turbot	Inactivated	Low	(90)
	Immersion	Immersion	Turbot	Inactivated	Low	(90)
*Aeromonas hydrophila*	Injection	Injection	Common carp	Inactivated	Moderate	(91)
*Streptococcus agalactiae*	Spray	Spray	Red tilapia	Inactivated	Moderate	(92)
*Vibrio anguillarum*	Immersion	Immersion	Sea bass	Live attenuated	Moderate	(93)
	Immersion	Injection	Sea bass	Live attenuated	High	(93)
	Oral	Immersion	Japanese flounder	Live attenuated	High	(94)
	Immersion	Immersion	Sea bass	Live attenuated	High	(95)
*Vibrio anguillarum*	Bath	Injection	Atlantic cod	Inactivated	High	(96)
*Edwardsiella tarda*	Oral	Immersion	Japanese flounder	Live attenuated	High	(97)
	Injection	Injection	Japanese flounder	Live attenuated	High	(98)
*Edwardsiella ictaluri*	Immersion	Oral	Vietnamese catfish	Inactivated	Moderate	(60)
*Flavobacterium columnare*	Immersion	Immersion	Nile tilapia	Inactivated	Antibody	(99)
	Immersion	Immersion	Channel catfish	Live attenuated	Moderate	(100)
*Lactococcus garvieae*	Injection	Oral	Trout	Inactivated	Moderate	(101)
*Lactococcus garvieae*	Oral	Oral	Trout	Inactivated	Moderate	(102)

**Table 4:** **Prime-boost vaccination strategies for viral diseases explored under experimental conditions in finfish**.

Disease	Prime delivery	Boost delivery	Fish species	Immunogenic protein	Antigen	Protection	Reference
Viral hemorrhagic septicemia	Oral	Oral	Olive flounder	Recombinant protein	Live	High	(4)
Infectious pancreatic necrosis	Injection	Oral	Atlantic salmon	Inactivated whole virus	Inactivated	ND*	(103)
Infectious salmon anemia virus	Injection	Oral	Atlantic salmon	Inactivated whole virus	Inactivated	ND*	(104)
Grouper iridovirus	Injection	Injection	Asian grouper	ORF-DNA recombinants	Plasmid DNA	Low	(105)
Spring virus of carp virus	Oral	Oral	Carp	Recombinant G-protein	Plasmid DNA	Moderate	(106)
Koi herpesvirus	Oral	Oral	Carp	ORF81 protein	Plasmid DNA	Moderate	(106)
Herpesviral hematopoietic necrosis virus	Injection	Immersion	Ryukin gold fish	Virus-CyHV-2	Inactivated	Moderate	(107)

*ND = Not done

### Heterologous Prime-Boost Vaccination Strategies

In the heterologous prime-boost strategies, the vector encoding the antigen used in the primary vaccine is different from the vector encoding the same antigen used in the booster vaccine. DNA vaccines combined with attenuated viral vectors, particularly those not able to cause disease in the target host species, have proved to be an effective combination for heterologous prime-boost vaccinations in mammals (108). The common viral vectors used in mammals are fowlpox virus (FPV) (109) and modified vaccinia virus Ankara strain (MVA) (108, 110). The DNA prime/FPV boost strategy was first demonstrated in influenza vaccinations using the hemagglutinin (HA) antigen, which resulted in high levels of anti-HA antibodies, IFNγ, and Th1 responses (108, 111). Similarly, a DNA prime/FPV boost regime using the HIV antigen produced high CD4^+^ and CD8^+^ levels that significantly reduced the HIV viral loads in macaque monkeys. Jia et al. (112) primed mice with an attenuated *Franciella tularensis* live vaccine strain capB mutant followed by a recombinant attenuated *Listeria monocytogenes* vector expressing the same *F. tularensis* antigen resulting in high levels of IFNγ, TNFα, IL-2, CD4^+^, and CD8^+^ cells. These studies show that heterologous prime-boost vaccination strategies enhance CMI responses, apart from increasing the antibody responses (87, 108, 113, 114). This is supported by observations made by Woodberry et al. (114) who showed that the ability of DNA prime/MVA boost regimes to increase CD8^+^ responses in an influenza challenge model was a sum of responses induced by the DNA prime vaccine alone plus the MVA boost. As pointed out by Woodland (87), increased CD8^+^ responses induced by heterologous prime-boost regimes increase CMI levels to protective thresholds in vaccinated animals. Although DNA vaccines have been studied in finfish (Table 4), there are no studies based on DNA prime/viral vector boost regimes shown to enhance CMI responses in finfish. Table 4 shows that most prime-boost vaccination regimes using DNA vaccines alone, without the boost of heterologous vectors expressing the same antigen encoded in DNA vaccines, are not highly protective in finfish. Therefore, there is a need to design heterologous prime-boost vaccination regimes able to enhance both CMI and humoral immune responses in finfish.

## General Discussion and Conclusion

In this review, we have shown that challenges that limit our success for designing highly protective mucosal vaccines are centered on optimizing mucosal vaccine design and host immune response factors. Factors that pose a challenge in optimizing mucosal vaccine design for finfish include (i) optimization of antigen doses in order to pave way for identifying the correlates of protective immunity on mucosal surfaces and protection against systemic pathogen dissemination; (ii) choice of antigen delivery systems (modalities) in which mucosal vaccines can be delivered as replicative or non-replicative antigens; (iii) protecting oral vaccines against degradation in the acidic environment of the gut; (iv) identifying the most potent adjuvants having highly effective immunostimulants able to enhance antigen uptake, processing, and presentation to cells of the adaptive immune system; (v) identifying the most effective vaccine delivery route able to produce highly protective mucosal and systemic antibody responses in vaccinated fish; and (vi) designing highly protective prime-boost vaccination strategies able to produce long-term protective immunity in vaccinated fish.

Host immune response factors that pose a challenge in designing protective mucosal vaccines for finfish include (i) oral tolerance – although the underlying mechanisms causing this condition in finfish have not been studied in detail, based on the limited studies carried out this far indications are that prolonged exposure to oral vaccination reduces the induction of systemic antibody responses in vaccinated fish; (ii) species variations need tailor-made vaccines to cope with individual species requirements; (iii) lack of established correlates of protective immunity required to determine the antigen dose able to prevent pathogen entry on mucosal surfaces and to prevent systemic pathogen dissemination for mucosal vaccines; and (iv) a complex host genomic response to mucosal vaccination whose network pathways can be difficult in identifying the most relevant genes required for optimization of vaccine performance in finfish.

Although there might be several other factors that limit the design of protective mucosal vaccines for finfish, this review highlights some of the most significant challenges currently hindering our ability to develop highly efficacious mucosal vaccines for finfish. Therefore, future studies should seek to overcome some of the challenges put forth in this review in order to pave way for the design of protective mucosal vaccines for finfish.

## Conflict of Interest Statement

The authors declare that the research was conducted in the absence of any commercial or financial relationships that could be construed as a potential conflict of interest.
